# Surgical and oncological efficacy of laparoscopic-assisted total gastrectomy versus open total gastrectomy for gastric cancer by propensity score matching: a retrospective comparative study

**DOI:** 10.1007/s00432-020-03503-4

**Published:** 2021-01-07

**Authors:** Yingcong Fan, Maoxing Liu, Shijie Li, Jianhong Yu, Xinyu Qi, Fei Tan, Kai Xu, Nan Zhang, Zhendan Yao, Hong Yang, Chenghai Zhang, Jiadi Xing, Zaozao Wang, Ming Cui, Xiangqian Su

**Affiliations:** 1 Key Laboratory of Carcinogenesis and Translational Research (Ministry of Education), Department of Gastrointestinal Surgery IV, Peking University Cancer Hospital and Institute, Beijing, 100142 China; 2 Key Laboratory of Carcinogenesis and Translational Research (Ministry of Education), Department of Endoscopy, Peking University Cancer Hospital and Institute, Beijing, 100142 China

**Keywords:** Gastric cancer, Laparoscopic-assisted total gastrectomy, Open total gastrectomy, Surgical safety, Oncological efficacy, Propensity score matching

## Abstract

**Purpose:**

The application of laparoscopic-assisted total gastrectomy (LATG) for resectable gastric cancer (GC) remains controversial compared with open total gastrectomy (OTG), especially for advanced gastric cancer (AGC) patients according to the inconsistent results demonstrated in the previous studies. The aim of this study was to evaluate the short-term and long-term outcomes between LATG and OTG in a population with more than 80% AGC patients by applying propensity score matching (PSM) method.

**Methods:**

The data of 365 clinical stage I–III GC cases who underwent total gastrectomy with D2 lymphadenectomy were retrospectively collected from January 2011 to April 2018 in the Department of Gastrointestinal Surgery IV of Peking University Cancer Hospital. Propensity scores were generated through taking all covariates into consideration and 131 pairs of patients receiving either LATG or OTG were matched. Intraoperative, postoperative, and survival parameters were compared in the matched groups accordingly. Risk factors for postoperative complications and overall survival were further analyzed.

**Results:**

Patient characteristics in the LATG and OTG groups were well balanced after PSM. LATG showed advantages with respect to shorter time to ambulation, first flatus, and first whole liquid diet intake. No significant differences were found between the two groups with regard to postoperative complications as well as overall survival in terms of different pathological stage. Older age was found as an independent risk factor for postoperative complications, and pathological stage for overall survival as well.

**Conclusion:**

LATG appears to have comparable surgical and oncological safety with OTG by experienced surgeons.

## Introduction

Despite a slight drop of incidence, gastric cancer (GC) remains the fifth most common malignancy and the third main causes of cancer death worldwide (Bray et al. 2018). Radical surgery plays an essential role in GC treatment for resectable cases. In the recent years, minimally invasive surgery has gained popularity due to its less invasive nature and its faster postoperative recovery (Herrera-Almario and Strong 2016; Pugliese et al. 2010). To date, several international cooperative multicenter randomized controlled trials (RCT) have demonstrated the short-term safety and long-term efficacy of laparoscopic-assisted distal gastrectomy (LADG) for distal GC (Hiki et al. 2018; Hu et al. 2016; Katai et al. 2010; Kim et al. 2016; Park et al. 2018; Wang et al. 2019; Yu et al. 2019). In many experienced institutions, laparoscopic surgery is a mature technique and optional approach, instead of conventional open surgery, for distal gastrectomy.

Nevertheless, compared with LADG, studies which provide convincing data to prove the feasibility and safety of laparoscopic-assisted total gastrectomy (LATG) remain insufficient. JCOG1401 and KLASS03 were two single-arm multicenter prospective studies conducted in Japan and Korea which aimed to evaluate the surgical safety of laparoscopic total gastrectomy (LTG) or laparoscopic-assisted total/proximal gastrectomy (LATG/LAPG) for patients with clinical stage I GC (Hyung et al. 2019; Kataoka et al. 2016). While the postoperative complication rates differed greatly. JCOG1401 trial reported a 29.1% incidence of in-hospital grade III–IV adverse events, which was really higher than KLASS03 study with 9.4% grade III or higher complication rate (Hyung et al. 2019; Katai et al. 2019). Owing to the difficulty of standard systematic D2 lymphadenectomy and esophagojejunostomy in total gastrectomy (TG), the risk of postoperative complications for advanced gastric cancer (AGC) patients underwent LATG may be even higher, while no RCTs have been launched to evaluate the feasibility and oncological efficacy of LATG for AGC till now. Besides, the results from retrospective studies also reported contradictory results. Although some studies demonstrated that LATG was associated with better intraoperative parameters and lower incidence of postoperative complications (Etoh et al. 2018; Lin et al. 2017), other studies found anastomotic leakage occurred more frequently in LATG than OTG group, even after taking the effect of learning curve into consideration (Sakamoto et al. 2019; Shim et al. 2013). More studies are still needed to confirm the non inferiority of LATG to OTG, especially for AGC patients. Therefore, we retrospectively investigated data from 365 patients with a majority of AGC cases who underwent LATG or OTG in the Department of Gastrointestinal Surgery IV of Peking University Cancer Hospital, comprehensively analyzed the data by using propensity score matching (PSM) method, and reported surgical safety as well as oncological efficacy between the two groups, in addition with risk factors for post-operative complications and overall survival.

## Patients and methods

### Patients

**Fig. 1:**
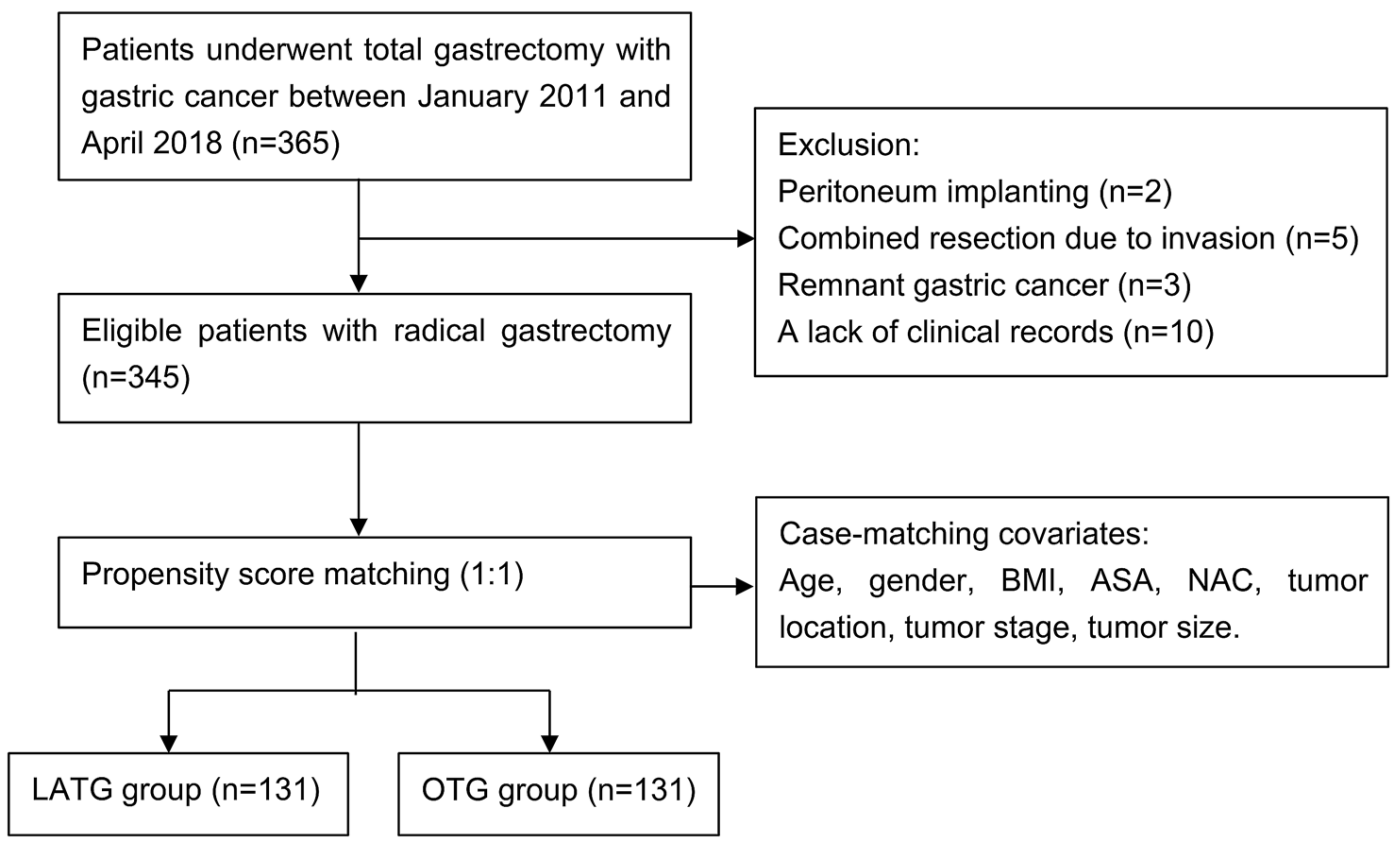
Study flow diagram. 365 patients were diagnosed with gastric cancer. 345 cases met the inclusion and exclusion criteria. After propensity score matching (PSM), there were 131 cases in each group. *LATG* laparoscopic-assisted total gastrectomy, *OTG* open total gastrectomy, *BMI* body mass index, *ASA* American Society of Anesthesiologist, *NAC* neoadjuvant chemotherapy

We retrospectively collected data from 365 patients who were diagnosed as clinical stage I–III GC and received radical TG between January 2011 and April 2018. The exclusive criteria were as follows: (1) peritoneum implanting confirmed in surgery (*n* = 2); (2) combined resection due to tumor invasion (*n* = 5); (3) remnant gastric cancer (*n* = 3); (4) incomplete clinical records (*n* = 10). The remaining 345 cases, with 198 and 147 cases undergoing LATG and OTG, respectively, were involved in this analysis. Then, 131 pairs of patients were matched from the two groups after PSM (Fig. 1 study flow diagram).

The clinicopathological characteristics were collected from surgical and nursing records as well as pathological reports. Diagnosis of GC along with clinical TNM stage were confirmed before surgery based on the preoperative examinations, including esophagogastroduodenoscopy with biopsy, chest, and abdominal CT scan, together with endoscopic ultrasonography. Comorbidities according to the American Society of Anesthesiologist (ASA) system were scored to evaluate the general condition of patients (Davenport et al. 2006). Clinical I–III stage GC patients with tumors located in the upper, middle, or whole stomach had undergone radical TG with standard D2 lymph node dissection, in line with the Japanese Gastric Cancer Treatment Guidelines (Japanese Gastric Cancer Association 2017). Besides, 37 patients received neo-adjuvant chemotherapy (NAC) with Xelox or Sox regimens before radical surgery due to their locally advanced clinical stage. Pathological stage was diagnosed according to the 8th Union for International Cancer Control (UICC)/American Joint Committee on Cancer (AJCC) staging system (Sano et al. 2017) after surgery. Patients with pathological stage ≥ pT2 or pN (positive) were treated with Xelox or Sox regimens for at least 4–8 cycles as adjuvant chemotherapy. Patients were well informed about the advantages and disadvantages of LATG and OTG before making their willing choices. Informed consent was obtained from each patient. This program was approved by the Research Ethics Committee of Peking University Cancer Hospital.

### Operative techniques

Radical gastrectomy with standard D2 regional lymphadenectomy was performed in line with the Japanese Gastric Cancer Treatment Guidelines (Japanese Gastric Cancer Association 2017). Roux-en-Y esophagojejunostomy reconstruction was routinely performed in surgery. Splenic hilar lymph node dissection was not routinely performed. For LATG, five trocars were inserted with unrestricted location. Gastrectomy and lymph nodes dissection were performed under the assistance of laparoscopy. Then, an incision about 5–7 cm was made in the midline of the upper abdomen to perform the esophagojejunostomy reconstruction procedure. In contrast, an incision about 15–20 cm by laparotomy was required in the performance of OTG surgery from start to finish. All the operations were conducted by the same experienced surgeon together with two assistants, who had already conducted at least 50 cases of LATG and OTG operations, respectively.

### Definitions

Postoperative morbidity and mortality were analyzed within 30 days after surgery. The postoperative complications, graded by the Clavien–Dindo classification system (Dindo et al. 2004), were divided into surgical related and non-surgical related complications. Surgical related complications were composed of pancreatic leakage, intra-abdominal bleeding, anastomotic bleeding, surgical site infection, seroperitoneum, intra-abdominal abscess, intestinal obstruction, intestinal fistula, anastomotic leakage, and lymphorrhagia. Although pulmonary infection, pleural effusion, cardiovascular dysfunction, and other problems comprised non surgical related complications. The overall survival was defined as the period from surgery to death or final follow-up date.

### Follow-up

The follow-up staffs in our department conducted regular follow-up and managed postoperative re-examination for patients at indicated time point, namely every 3 months for the first 2 years, 6 months for the following 3 years and 12 months after the first 5 years. All patients were followed until death or July 2020, ranging from 3 to 91 months.

### Propensity score matching and statistical analysis

To minimize the effect of selection bias of non randomized trial, PSM method was applied to balance the unevenly distributed patient baseline characteristics in the present study. Individual propensity scores were generated through a logistic regression model which included the following covariates: age, gender, body mass index (BMI), ASA score, NAC, macroscopic appearance, tumor location, tumor size, histological grade, and pathological TNM (pTNM) stage. Then, patients undergoing LATG or OTG were paired by a 1:1 nearest available score matching algorithm with a caliper width of 0.02 (Austin 2009; Ralph 1998; Rosenbaum 1983). After matching, balanced distribution of each covariate between the two groups was confirmed. The matched LATG and OTG groups were compared subsequently to estimate treatment effect of different surgical approaches.

Values of continuous variables were represented as ${\overline{\text{X}}} \pm {\text{SD}}$, and values of categorical variables were represented as frequencies and percentages. Differences between groups were examined by using Student’s *t* test, *χ*^2^ test or Fisher’s exact test where appropriate. Logistic regression was used to figure out risk factors for postoperative complications in univariate and multivariate analyses. Kaplan–Meier and log-rank test were performed to identify the difference of overall survival between the two groups according to different pathological stages. Cox proportional hazards regression model was used to determine the risk factors for survival. The items with *P* values less than 0.1 in the univariate analysis were adopted in the multivariate analysis in both regression equations. *P* values less than 0.05, derived from two-tailed tests, were considered statistically significant. All statistical analyses were performed with SPSS v. 25.0 (SPSS Inc., Chicago, IL, USA).

## Results

### Patients characteristics

The clinicopathological characteristics of patients receiving TG, both before and after PSM, were presented in Table 1. Before PSM, there were 345 patients in total, including 198 LATG cases and 147 OTG cases. There were significant differences between the two groups for the following patient characteristics: BMI (*P* = 0.040), NAC (*P* = 0.028) and tumor size (*P* = 0.040). After PSM, both LATG and OTG groups consisted of 131 patients each, and all the baseline parameters were well balanced between the two groups.

**Table 1:** Comparison of clinicopathological features of LATG and OTG groups

Characteristics	All patients (*n* = 345)		*P*	Patients after propensity matching (*n* = 262)		*P*
	LATG (*n* = 198)	OTG (*n* = 147)		LATG (*n* = 131)	OTG (*n* = 131)	
Age (year) *n* (%)			0.727			0.711
< 60	94 (47.5)	67 (45.6)		59 (45.0)	62 (47.3)	
≥ 60	104 (52.5)	80 (54.4)		72 (55.0)	69 (52.7)	
Gender *n* (%)			0.327			0.887
Female	41 (20.7)	37 (25.2)		33 (25.2)	34 (26.0)	
Male	157 (79.3)	110 (74.8)		98 (74.8)	97 (74.0)	
BMI (kg/m^2^) *n* (%)			***0.040***			0.409
< 30	195 (98.5)	139 (94.6)		129 (98.5)	127 (96.9)	
≥ 30	3 (1.5)	8 (5.4)		2 (1.5)	4 (3.1)	
NAC *n* (%)			***0.028***			0.679
No	183 (92.4)	125 (85.0)		117 (89.3)	119 (90.8)	
Yes	15 (7.6)	22 (15.0)		14 (10.7)	12 (9.2)	
ASA score *n* (%)			0.727			1.000
< 3	184 (92.9)	138 (93.9)		122 (93.1)	122 (93.1)	
≥ 3	14 (7.1)	9 (6.1)		9 (6.9)	9 (6.9)	
Macroscopic appearance *n* (%)			0.086			0.679
Non-linitis plastica	170 (85.9)	135 (91.8)		117 (89.3)	119 (90.8)	
Linitis plastica	28 (14.1)	12 (8.2)		14 (10.7)	12 (9.2)	
Tumor location *n* (%)			0.228			0.714
Upper	132 (66.7)	104 (70.7)		93 (71.0)	90 (68.7)	
Middle	38 (19.2)	31 (21.1)		24 (18.3)	29 (22.1)	
Total	28 (14.1)	12 (8.2)		14 (10.7)	12 (9.2)	
Tumor size (cm)						
Long diameter	4.9 ± 2.9	4.5 ± 2.5	0.457	4.6 ± 2.6	4.7 ± 2.6	0.867
Short diameter	1.9 ± 2.3	1.4 ± 1.4	***0.040***	1.4 ± 1.5	1.5 ± 1.5	0.462
Histologic type *n* (%)			0.222			0.669
Well differentiated	5 (2.5)	4 (2.7)		2 (1.5)	4 (3.1)	
Moderate	108 (54.5)	70 (47.6)		66 (50.4)	67 (51.1)	
Poor	85 (42.9)	72 (49.0)		63 (48.1)	59 (45.0)	
Undifferentiated	0 (0)	1 (0.7)		0 (0)	1 (0.8)	
pT stage *n* (%)			0.819			0.485
T1	23 (11.6)	18 (12.2)		12 (9.2)	16 (12.2)	
T2	25 (12.6)	15 (10.2)		20 (15.3)	13 (9.9)	
T3	84 (42.4)	64 (43.5)		59 (45.0)	55 (42.0)	
T4	66 (33.4)	50 (34.1)		40 (30.5)	47 (35.9)	
pN stage *n* (%)			0.187			0.651
N0	83 (41.9)	49 (33.3)		48 (36.6)	44 (33.6)	
N1	29 (14.6)	22 (15.0)		19 (14.5)	19 (14.5)	
N2	39 (19.7)	39 (26.5)		31 (23.7)	34 (26.0)	
N3	47 (23.7)	37 (25.2)		33 (25.2)	34 (26.0)	
pTNM stage *n* (%)			0.435			0.422
IA	22 (11.1)	15 (10.2)		14 (10.7)	11 (8.4)	
IB	16 (8.1)	13 (8.8)		10 (7.6)	12 (9.2)	
IIA	40 (20.2)	23 (15.6)		25 (19.1)	20 (15.3)	
IIB	30 (15.2)	17 (11.6)		16 (12.2)	20 (15.3)	
IIIA	41 (20.7)	42 (28.6)		28 (21.4)	39 (29.8)	
IIIB	31 (15.7)	18 (12.2)		24 (18.3)	14 (10.7)	
IIIC	18 (9.1)	19 (12.9)		14 (10.7)	15 (11.5)	

Bold and italic values are statistically significant *p * < 0.05

*LATG* laparoscopic-assisted total gastrectomy, *OTG* open total gastrectomy, *BMI* body mass index, *ASA* American Society of Anesthesiologist, *NAC* neoadjuvant chemotherapy

### Intraoperative and recovery outcomes

Before PSM, no significant differences were found between the two groups. After PSM, the LATG group showed significant advantages in the following items: reduced time to ambulation (21.9 vs. 24.9 h, *P* = 0.016), faster first flatus (2.8 vs. 3.3 days, *P* = 0.013), and shorter time to whole liquid diet intake (9.1 vs. 10.6 days, *P* = 0.048) (Table 2). In addition, LATG group showed less blood loss in surgery (67.3 ± 45.9 vs. 99.2 ± 119.1 ml, *P* = 0.102) compared with OTG group, although without significant statistical difference.

**Table 2:** Intra-operative and recovery outcomes of LATG and OTG groups

Variables	All patients (*n* = 345)		*P*	Patients after propensity matching (*n* = 262)		*P*
	LATG (*n* = 198)	OTG (*n* = 147)		LATG (*n* = 131)	OTG (*n* = 131)	
Operation time (min)	258.6 ± 55.8	257.7 ± 54.4	0.801	253.7 ± 50.9	257.5 ± 55.9	0.940
Blood loss (ml)	81.6 ± 69.2	99.5 ± 118.8	0.962	67.3 ± 45.9	99.2 ± 119.1	0.102
No. of dissected lymph nodes	32.6 ± 11.8	33.5 ± 13.4	0.545	33.2 ± 12.7	34.2 ± 13.6	0.211
Time to ambulation (h)	23.7 ± 20.5	24.6 ± 19.6	0.087	21.9 ± 15.3	24.9 ± 20.2	***0.016***
Time to resume bowel sound (D)	3.8 ± 1.5	3.6 ± 1.4	0.262	3.5 ± 1.3	3.6 ± 1.4	0.497
Time to first flatus (D)	3.1 ± 1.1	3.3 ± 1.0	0.537	2.8 ± 0.9	3.3 ± 1.0	***0.013***
Time to whole liquid diet (D)	9.3 ± 3.1	10.4 ± 6.6	0.198	9.1 ± 3.0	10.6 ± 6.9	***0.048***
Postoperative hospital stay (D)	11.7 ± 6.3	11.1 ± 5.3	0.441	11.6 ± 7.1	11.2 ± 5.5	0.814

Bold and italic values are statistically significant *p * < 0.05

*LATG* laparoscopic-assisted total gastrectomy, *OTG* open total gastrectomy, *D* days,

### Postoperative complications

Both before and after PSM, no significant differences were found between the groups in terms of postoperative adverse events (Table 3). After PSM, the total number of postoperative complications were 28 (21.4%) and 20 (15.3%) in the LATG and OTG group respectively, including surgical related complications (19, 14.5% vs. 11, 8.4%, *P* = 0.121) and the non surgical related complications (15, 11.5% vs. 12, 9.2%, *P* = 0.542). The incidence of severe complications showed no significant statistical difference, which referred to the complication severity no less than Grade III according to the Clavien–Dindo classification system, between the two groups.

**Table 3:** Postoperative complications of LATG and OTG groups

Variable	All patients (*n* = 345)		*P*	Patients after propensity matching (*n* = 262)		*P*
	LATG (*n* = 198)	OTG (*n* = 147)		LATG (*n* = 131)	OTG (*n* = 131)	
Total complications *n* (%)	42 (21.2)	24 (16.3)	0.254	28 (21.4)	20 (15.3)	0.201
Surgical related *n* (%)	28 (14.1)	13 (8.8)	0.133	19 (14.5)	11 (8.4)	0.121
Pancreatic leakage	1 (0.5)	2 (1.4)	0.577	1 (0.8)	2 (1.5)	1.000
Intra-abdominal bleeding	5 (2.5)	0 (0)	0.074	3 (2.3)	0 (0)	0.247
Anastomotic bleeding	6 (3.0)	2 (1.4)	0.475	5 (3.8)	2 (1.5)	0.447
Surgical site infection	1 (0.5)	2 (1.4)	0.577	1 (0.8)	2 (1.5)	1.000
Seroperitoneum	4 (2.0)	1 (0.7)	0.399	2 (1.5)	1 (0.8)	1.000
Intra-abdominal abscess	3 (1.5)	1 (0.7)	0.639	2 (1.5)	1 (0.8)	1.000
Intestinal obstruction	2 (1.0)	1 (0.7)	1.000	1 (0.8)	0 (0)	1.000
Intestinal fistula	3 (1.5)	2 (1.4)	1.000	3 (2.3)	2 (1.5)	1.000
Anastomotic leakage	8 (4.0)	2 (1.4)	0.199	6 (4.6)	1 (0.8)	0.120
Lymphorrhagia	4 (2.0)	0 (0)	0.139	2 (1.5)	0 (0)	0.498
Non-surgical related *n* (%)	23 (11.6)	15 (10.2)	0.679	15 (11.5)	12 (9.2)	0.542
Pulmonary infection	16 (8.1)	13 (8.8)	0.801	11 (8.4)	10 (7.6)	0.820
Pleural effusion	17 (8.6)	9 (6.1)	0.391	9 (6.9)	6 (4.6)	0.425
Cardiovascular system	3 (1.5)	1 (0.7)	0.639	3 (2.3)	0 (0)	0.247
Others	0 (0)	1 (0.7)	0.184	20 (15.3)	18 (13.7)	0.213
Clavien-Dindo classification *n* (%)						
< 3	30 (15.2)	21 (14.3)	0.823	20 (15.3)	18 (13.7)	0.726
≥ 3	12 (6.1)	3 (2.0)	0.070	8 (6.1)	2 (1.5)	0.053

*LATG* laparoscopic-assisted total gastrectomy, *OTG* open total gastrectomy

### Univariate and multivariate analysis of risk factors associated with postoperative complications

Univariate and multivariate logistic regression analyses were performed to analyze the risk factors for postoperative complications among the matched cases. In the univariate analysis, age (*P* = 0.027), gender (*P* = 0.059), operation time (*P* = 0.005), and blood loss in surgery (*P* = 0.031) were closely associated with postoperative morbidity. In the multivariate analysis, age (*P* = 0.038) was identified as an independent risk factor for postoperative complications (Table 4).

**Table 4:** Univariate and multivariate analyses of risk factors of postoperative complications after PSM

Variables	Postoperative complications		Univariate analysis *P*	Multivariate analysis		
	No (*n* = 214)	Yes (*n* = 48)		OR	95% CI	*P*
Surgical approach *n* (%)						
OTG	111 (51.9)	20 (41.7)				
LATG	103 (48.1)	28 (58.3)	0.203			
Age (years) *n* (%)						
< 60	105 (49.1)	15 (31.2)		1 [Reference]		
≥ 60	109 (50.9)	33 (68.8)	***0.027***	2.082	1.042–4.160	***0.038***
Gender *n* (%)						
Female	154 (72.0)	7 (14.6)		1 [Reference]		
Male	60 (28.0)	41 (85.4)	0.059	1.790	0.728–4.403	0.205
BMI *n* (%)						
< 30	209 (97.7)	47 (97.9)				
≥ 30	5 (2.3)	1 (2.1)	0.916			
ASA *n* (%)						
< 3	198 (92.5)	46 (95.8)				
≥ 3	16 (7.5)	2 (4.2)	0.419			
NAC *n* (%)						
No	191 (89.3)	45 (93.8)				
Yes	23 (10.7)	3 (6.3)	0.352			
pT stage *n* (%)^a^						
T1	24 (11.2)	4 (8.3)				
T2	27 (12.6)	6 (12.5)	0.683			
T3	93 (43.5)	21 (43.8)	0.608			
T4	70 (32.7)	17 (35.4)	0.533			
pN stage *n* (%)^a^						
N0	76 (35.5)	16 (33.3)				
N1	32 (15.0)	6 (12.5)	0.825			
N2	49 (22.9)	16 (33.3)	0.270			
N3	57 (26.6)	10 (20.8)	0.678			
pTNM *n* (%)^a^						
I	35 (16.4)	12 (25.0)				
II	70 (32.7)	11 (22.9)	0.094			
III	109 (50.9)	25 (52.1)	0.316			
Macroscopic appearance *n* (%)						
Non-linitis plastica	191 (89.3)	45 (93.8)				
Linitis plastica	23 (10.7)	3 (6.3)	0.352			
Operative time (min)	251.1 ± 51.5	275.8 ± 57.6	***0.005***	1.006	1.000–1.012	0.054
Blood loss (ml)	76.9 ± 75.6	111.2 ± 139.8	***0.031***	1.003	0.999–1.006	0.106
Tumor size (cm)						
Long diameter	4.6 ± 2.6	4.8 ± 2.6	0.747			
Short diameter	1.4 ± 1.5	1.4 ± 1.2	0.930			
Tumor location *n* (%)^a^						
Upper	146 (68.2)	37 (77.1)				
Middle	45 (21.0)	8 (16.7)	0.405			
Total	23 (10.7)	3 (6.3)	0.300			
Histologic type *n* (%)^a^						
Well differentiated	4 (1.9)	2 (4.2)				
Moderate differentiated	109 (50.9)	24 (50.0)	0.359			
Poor differentiated	101 (47.2)	21 (43.8)	0.329			
Undifferentiated	0 (0)	1 (2.1)	1.000			
No. of comorbidities *n* (%)^a^						
0	159 (74.3)	35 (72.9)				
1	49 (22.9)	11 (22.9)	0.959			
2	6 (2.8)	2 (4.2)	0.620			

Bold and italic values are statistically significant *p * < 0.05

*LATG* laparoscopic-assisted total gastrectomy, *OTG* open total gastrectomy, *BMI* body mass index, *ASA* American Society of Anesthesiologist, *NAC* neoadjuvant chemotherapy, *OR* odds ratio, CI confidence interval,

^a^For categorical variable, each first item of classification was used as reference in the univariate logistic regression

### Survival

**Fig. 2:**
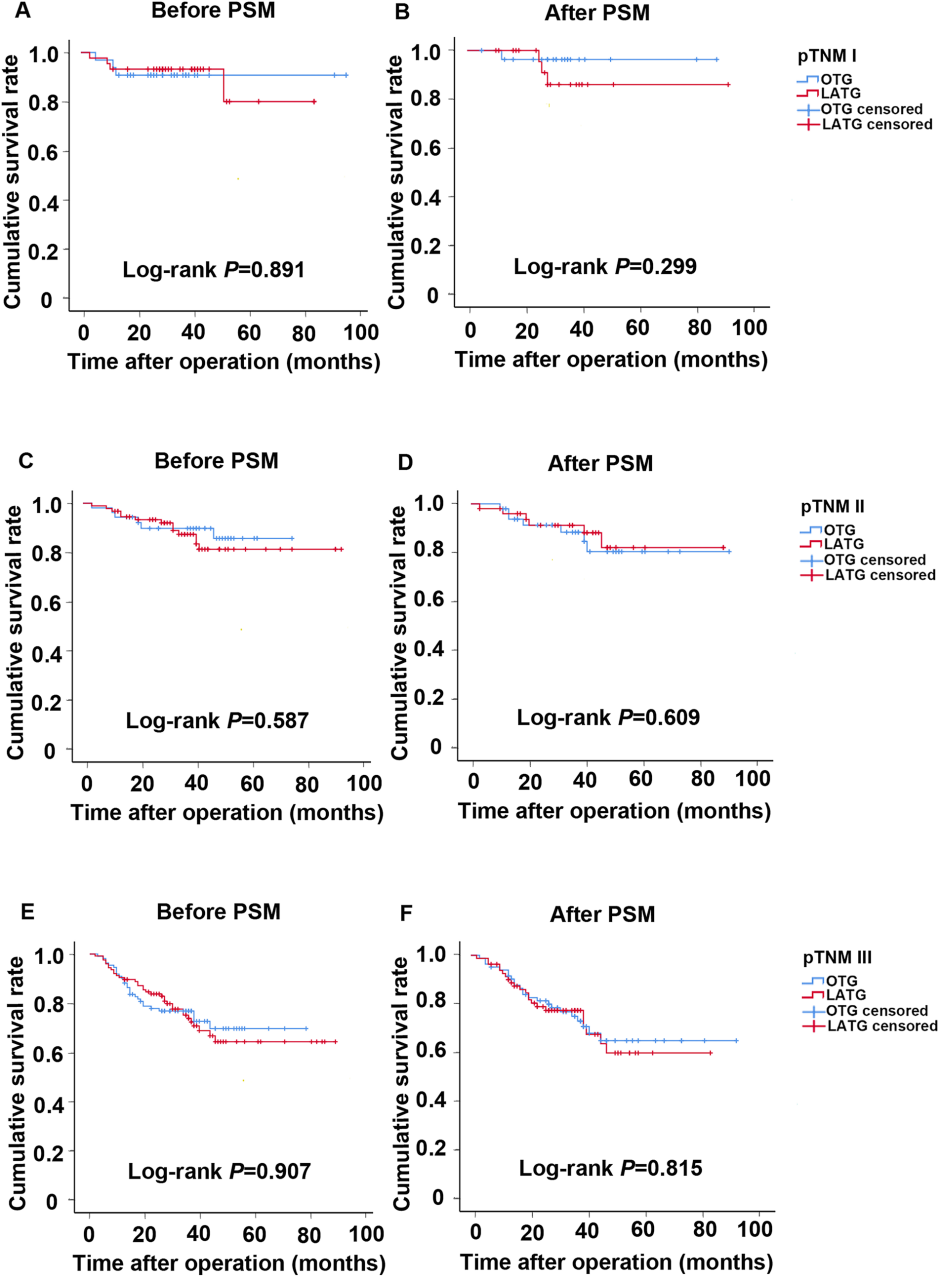
Cumulative survival rates of both LATG and OTG groups according to pathological stages (I, II, III). **a**, **b** The cumulative survival rates for pathological stage I were similar between LATG and OTG groups both before PSM (*P* = 0.891) and after PSM (*P* = 0.299). **c**, **d** The cumulative survival rates for pathological stage II were similar between LATG and OTG groups both before PSM (*P* = 0.587) and after PSM (*P* = 0.609). **e**, **f** The cumulative survival rates for pathological stage III were similar between LATG and OTG groups both before PSM (*P* = 0.907) and after PSM (*P* = 0.815). *LATG* laparoscopic-assisted total gastrectomy, *OTG* open total gastrectomy, *PSM* propensity score matching, *pTNM* pathological TNM stage

The overall survival of patients in LATG and OTG groups according to different pathological stages were presented by Kaplan–Meier curves both before and after PSM (Fig. 2). The median follow-up period was 36 (range 3–91) months. There were no significant differences between the LATG and OTG groups in terms of the same pathological stage. Before PSM, the cumulative survival rate between LATG and OTG in each stage was comparable (Stage I log-rank *P* = 0.891, Stage II log-rank *P* = 0.587, Stage III log-rank *P* = 0.907). After PSM, the overall survival rates were still similar between the two groups in each stage respectively (Stage I log-rank *P* = 0.299, Stage II log-rank *P* = 0.609, Stage III log-rank *P* = 0.815). However, the long-term survival rates differed significantly when it was stratified by different stages in either surgical types. Before PSM, the cumulative survival rates decreased gradually with the increased pathological stage of patients in both LATG and OTG groups (LATG log-rank *P* = 0.002, OTG log-rank *P* = 0.013). The results were similar in both groups after PSM (LATG log-rank *P* = 0.022, OTG log-rank *P* = 0.020), indicating a close correlation between survival and pathological stage (data not shown).

### Univariate and multivariate analysis of risk factors associated with overall survival

**Fig. 3:**
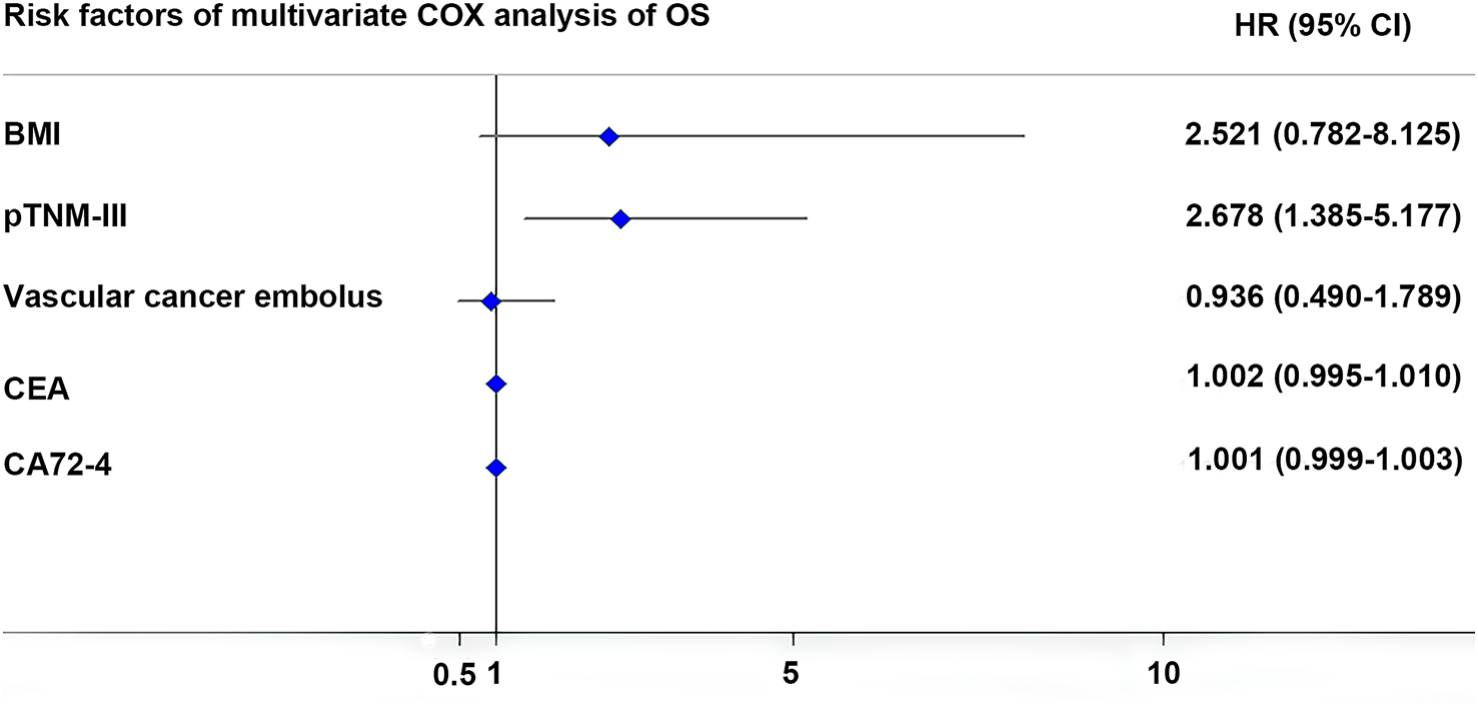
Forest graph of risk factors from multivariate COX analysis of overall survival. In the multivariate analysis, pTNM-III (*P* = 0.003) was significant risk factors of OS. *OS* overall survival, *HR* hazard ratio, *CI* confidence interval, *BMI* body mass index, *pTNM* pathological TNM stage, *CEA* carcinoembryonic antigen, *CA72-4* carbohydrate antigen 72–4

In the univariate analysis, we found BMI (*P* = 0.089), pTNM stage (*P* < 0.001), vascular cancer embolus (*P* = 0.069), CEA (*P* = 0.004) and CA72-4 (*P* = 0.002) were closely associated with overall survival among the matched cases (Table 5). Further, pTNM stage (*P* = 0.003) was identified as an independent risk factor for overall survival in the multivariate analysis, the hazard ratio of stage III was 2.678-fold as much as stage I/II, with 95% confidence interval of 1.385–5.177 (Fig. 3).

**Table 5:** Univariate and multivariate analyses of risk factors of overall survival after PSM

Variables	Univariate analysis		*P*	Multivariate analysis		*P*
	HR	95% CI		HR	95% CI	
Surgical procedure						
OTG	1 [Reference]					
LATG	0.969	0.589–1.594	0.901			
Age (years)						
< 60	1 [Reference]					
≥ 60	0.727	0.437–1.207	0.218			
Gender						
Female	1 [Reference]					
Male	0.980	0.548–1.754	0.946			
BMI						
< 30	1 [Reference]			1 [Reference]		
≥ 30	0.363	0.113–1.166	***0.089***	2.521	0.782–8.125	0.121
ASA						
< 3	1 [Reference]					
≥ 3	1.058	0.384–2.914	0.913			
NAC						
No	1 [Reference]					
Yes	0.749	0.340–1.647	0.472			
pTNM						
I/II	1 [Reference]			1 [Reference]		
III	2.836	1.623–4.957	< ***0.001***	2.678	1.385–5.177	***0.003***
Macroscopic appearance						
Non-linitis plastica	1 [Reference]					
Linitis plastica	0.618	0.294–1.300	0.204			
Blood loss in surgery (ml)	0.999	0.996–1.002	0.703			
Time of surgery (min)	1.002	0.997–1.006	0.501			
Postoperative complication						
No	1 [Reference]					
Yes	0.777	0.428–1.409	0.405			
Vascular cancer embolus						
(−)	1 [Reference]			1 [Reference]		
(+)	0.601	0.347–1.040	***0.069***	0.936	0.490–1.789	0.842
CEA (ng/ml)	1.006	1.002–1.010	***0.004***	1.002	0.995–1.010	0.572
CA199 (U/ml)	1.000	1.000–1.001	0.455			
CA72–4 (U/ml)	1.001	1.001–1.002	***0.002***	1.001	0.999–1.003	0.417

Bold and italic values are statistically significant *p * < 0.05

*LATG* laparoscopic-assisted total gastrectomy, *OTG* open total gastrectomy, *BMI* body mass index, *ASA* American Society of Anesthesiologist, *NAC* neoadjuvant chemotherapy, *CEA* carcinoembryonic antigen, *CA199* carbohydrate antigen 199, *CA72–4* carbohydrate antigen 72–4, *HR* hazard ratio, *CI* confidence interval

## Discussion

Studies indicate that laparoscopic radical gastrectomy is a feasible alternative or optional therapy for early stage of GC; it is less invasive and is associated with faster postoperative recovery than conventional open surgery (Sakuramoto et al. 2013; Zeng et al. 2012). A Korean nationwide KLASS-01 clinical trial demonstrated that LADG surgery was as safe as ODG surgery and had a lower rate of wound complications for stage I GC patients (Kim et al. 2016). The nationwide JCOG0912 study in Japan reached similar conclusion (Nakamura et al. 2013). Further, a Japanese nationwide JCOG0703 study confirmed the short-term safety and long-term efficiency of LADG, comparing with open surgery in stage I GC (Hiki et al. 2018; Katai et al. 2010). As for locally advanced gastric cancer, CLASS-01 and COACT 1001 trials indicated that experienced surgeons perform radical LADG safely and feasibly, and the latest follow-up data suggested a comparable 3-year DFS of LADG to ODG (Hu et al. 2016; Park et al. 2018; Wang et al. 2019; Yu et al. 2019).

However, owing to the extended lymph node dissection and complex digestive reconstruction that are performed in LATG, whether the surgical and oncological outcomes of LATG are not inferior to OTG remains controversial. When compared with LADG, whose standard D2 lymphadenectomy includes nos. 1, 3, 4sb, 4d, 5, 6, 7, 8a, 9, 11p, 12a, LATG requires additional resection of nos. 2, 4sa, and 11d lymph nodes. For the gastroesophageal junction adenocarcinoma invading the esophagus, resection of No. 110, 111, 19 and 20 lymph nodes are further required to obtain a sufficient resection margin (Japanese Gastric Cancer Association 2017). Moreover, considering the limited space, complex lymph node metastatic pathways and complicated vessels around the cardia, the lymph node dissection of lower mediastinum in LATG is much more challenging. In addition, esophagojejunostomy is even more difficult than gastrojejunostomy. The higher tension and less blood supply around the anastomosis after esophagojejunostomy might lead to higher risk of postoperative complications and poorer prognosis of LATG compared with LADG (Lee et al. 2012; Petersen et al. 2013; Shinohara et al. 2009). JCOG1401 has reported a 66.4% postoperative complication rate for in-hospital grade II–IV adverse events (Katai et al. 2019). Besides, several retrospective studies have demonstrated that increased anastomotic leakage and stenosis were more frequently observed in LATG (Sakamoto et al. 2019; Petersen et al. 2013). Esophagojejunal anastomotic leakage has been considered as one of the most serious complications after TG. It not only leads to immediate clinical consequences, such as prolonged hospital stay, increased mortality and elevated surgery-related costs, but also affects the long-term outcomes, including poorer quality of life, increased recurrence rate, and decreased overall survival (Kofoed et al. 2015; Nagasako et al. 2012; Sierzega et al. 2010; Yoo et al. 2011). Moreover, oncological safety seems not to be the concern of current prospective research, it was even not set as an endpoint in CLASS02 trial, whose primary and secondary endpoints were all focus on surgical safety between LTG and OTG groups for clinical stage I gastric cancer patients (He et al. 2018). In order to provide more clues to this inconclusive issue, retrospective data were collected and analyzed by using PSM in the present study.

Among the matched cases after PSM, the results demonstrated that the LATG group had a faster postoperative recovery compared with OTG group, especially in relation to the following measures: shorter interval to ambulation, faster first flatus, and reduced time to whole liquid diet intake. This result is in accordance with studies reporting favorable short-term outcomes of LADG surgery and highlights the advantages of minimally invasive surgery (Hu et al. 2016; Kim et al. 2016; Wang et al. 2019). Besides, no significant differences were observed in terms of postoperative adverse events. LATG had less cases with surgical site infection, which may be due to the shorter length of incision in laparoscopic surgery than that of open surgery.

In this study, a bit more cases of intra-abdominal bleeding and anastomotic bleeding were noted in LATG compared to OTG. The following reasons may lead to higher rate of intra-abdominal bleeding in LATG. First, physical techniques, such as compression or suturing could be directly performed in open surgery by surgeons, while such procedures are highly limited in laparoscopic surgery. Operators mainly rely on thermal surgical modalities and topical sealants, such as ultrasonic dissectors, hemostasis clip, and gelatin matrix to achieve hemostasis in laparoscopic surgery (Hang et al. 2015; Lattouf et al. 2007). Second, the use of ultrasonic scalpel other than hemostasis clip to cut and close the main arteries and veins may lead to delayed intra-abdominal bleeding (Szold et al. 2018). Third, open surgery tends to cause a higher degree of activation of the clotting system than laparoscopic surgery (Diamantis et al. 2007). Therefore, medical devices, such as matrix of bovine thrombin and collagen or gelatin, polymers of cellulose backed with sponge, are highly recommended to establish a stable network for the closure of the microvascular oozing in laparoscopy (Vecchio et al. 2016).

Anastomotic bleeding is relatively rare but lethal if not treated immediately. The use of gastrointestinal staplers was necessary to perform gastrointestinal anastomosis (Vecchio et al. 2016). However, the smaller size of the abdominal incision in laparoscopic surgery may cause difficulties to adjust the stapler’s angle in the esophagojejunostomy and lead to increased tension and potential bleeding risk in the anastomosis. In addition, a small incision makes it difficult to suture and reinforce the anastomosis, compared with conventional open surgery. Therefore, extra examination methods, such as intraoperative endoscopy examination or observation of the color of fluid draining through the gastrointestinal decompression tube and hand-sewing were recommended (Kim et al. 2012; Lee et al. 2017).

Anastomotic leakage is one of the main causes of mortality after surgery. In our study, the incidence of anastomotic leakage was higher in LATG than OTG group after PSM (4.6% vs. 0.8%, *P* = 0.120). Although without statistical significance, the results are consistent with the previous studies (Sakamoto et al. 2019; Petersen et al. 2013). The complication rate of anastomotic leakage has been reported to be around 1.5–7.4% in LATG (Etoh et al. 2018; Jeong et al. 2009; Lee et al. 2015). A Japanese nationwide inpatient database analysis study (Sakamoto et al. 2019) explained that the higher rate of anastomotic leakage in LATG was mainly due to the difficulty in laparoscopic esophagojejunostomy, which may cause increased tension and insufficient blood supply of the anastomosis. The two most serious cases in our study with anastomotic leakage, who underwent a second surgery for exploratory laparotomy and debridement or thoracotomy and debridement respectively, were mainly due to increased tension caused by the retraction of the location of anastomotic stoma, which went to the inferior mediastinum. Besides, the different types of esophagojejunostomy which could reduce the anastomotic leakage rates are needed in future studies (Athanasiou et al. 2019; Gong and Li 2017; Montenovo et al. 2011).

In the univariate and multivariate analyses, age turned out to be an independent risk factor for postoperative complications. Nowadays, aging issue has become an increasingly prominent problem around the whole world (Li et al. 2017; Pal et al. 2010). The elderly patients showed less tolerance to invasive treatments due to increased possibility of more comorbidities and reduced reserve capacity of organs. Some studies reported the elderly are highly associated with postoperative complications and unplanned reoperation (Jung et al. 2015; Li et al. 2017; Su et al. 2011). The application of LATG to elderly patients remains controversial and needs further studies.

With regard to the long-term survival, our data showed a comparable 5-year overall survival rate between LATG and OTG group in all subgroup analyses. Even for the highly advanced stage, especially stage III, the prognosis showed no significant difference between the laparoscopic group and open group. Therefore, long-term survival will not be influenced by surgical types in each pathological stage, which was in accordance with other studies (Lee et al. 2013; Lin et al. 2017). The independent risk factor for overall survival analyzed by multiple COX regression was pathological stage. Our results also indicated that GC patients of stage III had a higher risk to develop worse prognosis than stage I and II. Nowadays, with the application of adjuvant chemotherapy, which is routinely used for AGC, the prognosis of gastric cancer is better than before, while more studies are needed to achieve a better prognosis.

The main limitation of the present study was that it was a single center retrospective study with limited cases. Although the potential selection bias had been minimized using PSM, which increased the reliability of this study, a risk of underpowered statistical analysis could not be avoided because of the reduced sample size after PSM. Therefore, a multicenter RCT study is expected to draw more accurate and convincing conclusions.

## Conclusion

In conclusion, LATG performed by experienced surgeons could provide comparable surgical safety and survival outcomes with OTG for GC patients.

## Data Availability

The datasets used in this study are available from the corresponding author on reasonable request.
